# The Antiviral Molecule 5-Pyridoxolactone Identified Post BmNPV Infection of the Silkworm, *Bombyx mori*

**DOI:** 10.3390/ijms22147423

**Published:** 2021-07-10

**Authors:** Xiaoting Hua, Quan Zhang, Wei Xu, Xiaogang Wang, Fei Wang, Ping Zhao, Qingyou Xia

**Affiliations:** 1 Biological Science Research Center, Southwest University, Chongqing 400715, China; huaxiaotingswu@126.com (X.H.); zq308350576@outlook.com (Q.Z.); xuwei2721@163.com (W.X.); fwangswu@gmail.com (F.W.); zhaop@swu.edu.cn (P.Z.); 2 State Key Laboratory of Silkworm Genome Biology, Southwest University, Beibei, Chongqing 400715, China; 3 China Chongqing Key Laboratory of Chinese Medicine & Health Science, Chongqing Academay of Chinese Materia Medica, Chongqing 400065, China; wangyang217804@126.com; 4 Chongqing Key Laboratory of Sericultural Science, Chongqing Engineering and Technology Research Center for Novel Silk Materials, Southwest University, Chongqing 400715, China

**Keywords:** BmNPV, *Bombyx mori*, metabolomic, antiviral, 5-pyridoxolactone

## Abstract

*Bombyx mori* nucleopolyhedrovirus (BmNPV) is a pathogen that causes great economic losses in sericulture. Many genes play a role in viral infection of silkworms, but silkworm metabolism in response to BmNPV infection is unknown. We studied BmE cells infected with BmNPV. We performed liquid chromatography coupled with tandem mass spectrometry (LC-MS/MS)-based non-targeted metabolomics analysis of the cytosolic extract and identified 36, 76, 138, 101, 189, and 166 different molecules at 3, 6, 12, 24, 48, and 72 h post BmNPV infection (hpi) compared with 0 hpi. Compounds representing different areas of metabolism were increased in cells post BmNPV infection. These areas included purine metabolism, aminoacyl−tRNA biosynthesis, and ABC transporters. Glycerophosphocholine (GPC), 2-hydroxyadenine (2-OH-Ade), gamma-glutamylcysteine (γ-Glu-Cys), hydroxytolbutamide, and 5-pyridoxolactone glycerophosphocholine were continuously upregulated in BmE cells post BmNPV infection by heat map analysis. Only 5-pyridoxolactone was found to strongly inhibit the proliferation of BmNPV when it was used to treat BmE cells. Fewer infected cells were detected and the level of BmNPV DNA decreased with increasing 5-pyridoxolactone in a dose-dependent manner. The expression of BmNPV genes ie1, helicase, GP64, and VP39 in BmE cells treated with 5-pyridoxolactone were strongly inhibited in the BmNPV infection stage. This suggested that 5-pyridoxolactone may suppress the entry of BmNPV. The data in this study characterize the metabolism changes in BmNPV-infected cells. Further analysis of 5-pyridoxolactone, which is a robust antiviral molecule, may increase our understanding of antiviral immunity.

## 1. Introduction

Biochemical factors, including small molecules, can vary with development, metamorphosis, and immune responses in insects [1]. The silkworm, *Bombyx mori* L. (Lepidoptera: Bombycidae), has been domesticated for silk production for more than 5000 years. B. mori is important in the silk industry of China, India, and other developing countries, and is also a useful model for genetics and immunology studies [2,3,4,5]. *Bombyx mori* nucleopolyhedrovirus (BmNPV) baculovirus is a major pathogen of the silkworm and causes serious economic losses [6,7]. Genetics and molecular biology studies have generated antiviral strains and revealed immunological responses [8,9,10,11,12]. However, the resistance mechanism remains unclear.

Previous studies have explored the *B. mori* resistance mechanism using genome, transcriptome, or proteasome analysis. Using a genome-wide microarray in midgut tissue of the Indian silkworm (*B. mori*), many differential expressed genes were characterized in a resistant race (Sarupat) and a susceptible race [13]. Sagisaka et al. compared the transcriptome from silkworm ovary cell lines pre and post BmNPV infection. They found that the expression of BmEts, BmToll10-3, tetraspanin, MMPvali-ant1, and ABC transporter was increased while the expressions of HSP20 and HSP90 were reduced by BmNPV infection [14]. Comparison of fat body samples and midgut samples from susceptible and resistant silkworm strains by transcriptome analysis showed that the number of alternative splicing events in the BmNPV-susceptible silkworms was greater than that in the BmNPV-resistant silkworms [15]. Zhou et al. compared the transcriptomes of two silkworm lines differing in their resistance to BmNPV and identified differentially expressed genes including amino acid transporters, serine proteases, and serpins [16]. Wang et al. conducted a transcriptome study and found that cytochrome c (cytc) had a significant response to BmNPV infection [17]. In a comparative proteomics study, arginine kinase [18] was involved in the antiviral process of different resistant strains of silkworm and caspase-1 and serine protease could also be related to antiviral activities [19]. Many studies have identified the viral infection-related host genes but the metabolic responses during the infection of BmNPV in silkworm and small intracellular molecules from the silkworm that may suppress the proliferation of BmNPV are unreported.

Metabolomics is a high-throughput technique that can be used to study biological processes involving small molecules [20]. Several small intracellular molecules, such as cyclic 3′5′-adenosine monophosphate (AMP) or cyclic guanosine monophosphate (GMP), calcium, DAG, IP3, and reactive oxygen and nitrogen species (ROS, NOS), have been reported as second messengers and play roles in intracellular signaling pathways [20]. We previously characterized cGAMP from the BmE cells post BmNPV infection at different times. Based on the calibration curves of chemically synthesized cGAMP, we found that the concentrations of cytosolic cGAMP increased upon viral infection. This revealed the cGAMP–BmSTING pathway of the regulation of antiviral immunity in insects [10]. To further study intracellular small molecules involved in intracellular viral infection, the extraction method [21] of intracellular related small molecules was used and the cell extracts post BmNPV infection were analyzed by LC-MS/MS technology.

We used BmE cells infected with BmNPV and performed liquid chromatography coupled with tandem mass spectrometry (LC-MS/MS)-based non-targeted metabolomics analysis of the cytosolic extract. We identified 36, 76, 138, 101, 189, and 166 differential metabolites at 3, 6, 12, 24, 48, and 72 h post infection (hpi) compared with 0 hpi separately. Compounds that increased in the cells post BmNPV infection included those involved with purine metabolism, aminoacyl−tRNA biosynthesis, and ABC transporters. Glycerophosphocholine (GPC), 2-hydroxyadenine (2-OH-Ade), gamma-glutamylcysteine (γ-Glu-Cys), 4-hydroxytolbutamide, and 5-pyridoxolactone were continuously upregulated in BmE cells post BmNPV infection and screened for further analysis. BmE cells were treated with these small-molecule metabolites and BmNPV simultaneously. Only 5-pyridoxolactone strongly inhibited the proliferation of BmNPV. The amount of BmNPV DNA decreased significantly with increasing 5-pyridoxolactone in a dose-dependent manner.

## 2. Results

### 2.1. Metabolites in the BmE Cells Infected with BmNPV

**Figure 1:**
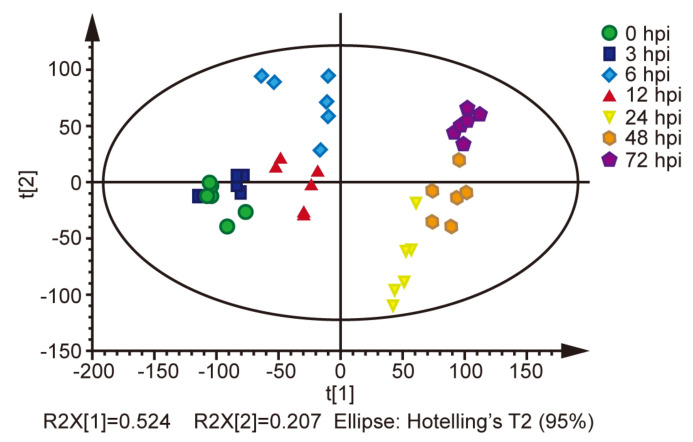
OPLS-DA of identified metabolites between samples (six biological replicates).

The metabolomics of BmE cells post BmNPV infection at 0, 3, 6, 12, 24, 48, and 72 hpi were analyzed by LC-MS/MS. A total of 265 metabolites were identified. Total ion chromatograms (TICs) of different samples are shown in Figure S1. Among the metabolites were amino acids, fatty acids, organic acids, sugars, sterols and lipids, and alkanes, with the remainder categorized as unclassified metabolites. OPLS-DA analysis showed a clear separation between samples except at 3 hpi. The difference in metabolic characters then increased and was sustained from 24 to 72 hpi (Figure 1). This result implies that the metabolic characters between these time points are different.

### 2.2. Differential Analysis of the Identified Metabolites Following BmNPV Stimulation

**Figure 2:**
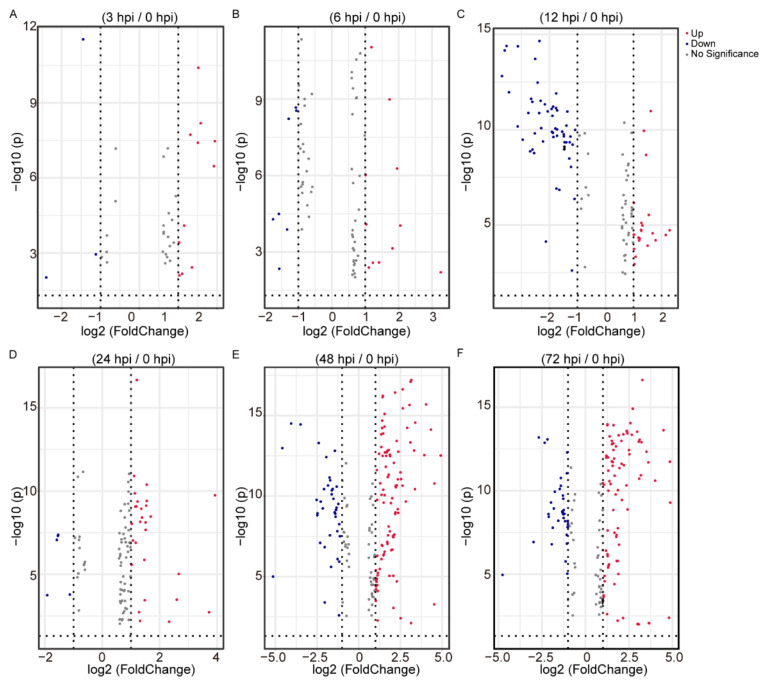
The level of metabolites in BmE cells stimulated with BmNPV for 3 h (3 hpi) compared with control (0 hpi) (**A**), 6 hpi with 0 hpi (**B**), 12 hpi with 0 hpi (**C**), 24 hpi with 0 hpi (**D**), 48 hpi with 0 hpi (**E**), and 72 hpi with 0 hpi (**F**). Differential metabolites were screened by fold change ≥2 and ≤0.5. Red plots represent upregulated metabolites, blue plots represent downregulated metabolites, and grey plots represent non-differential metabolites.

To highlight differences in the metabolites in BmE cells at different infection stages, differential analysis was performed using data from 0, 3, 6, 12, 24, 48, and 72 hpi. The 256 metabolites were identified separately in 3, 6, 12, 24, 48, and 72 hpi, when compared with 0 hpi. There were 11 upregulated and 3 downregulated metabolites at 3 hpi/0 hpi (Figure 2A, Table S3_1), 11 upregulated and 7 downregulated metabolites at 6 hpi/0 hpi (Figure 2B, Table S3_2), 23 upregulated and 56 downregulated metabolites at 12 hpi/0 hpi (Figure 2C, Table S3_3), 28 upregulated and 5 downregulated metabolites at 24 hpi/0 hpi (Figure 2D, Table S3_4), 100 upregulated and 39 downregulated metabolites at 48 hpi/0 hpi (Figure 2E, Table S3_5), and 88 upregulated and 34 downregulated metabolites at 72 hpi/0 hpi (Figure 2F, Table S3_6).

### 2.3. Pathway Analysis of the Identified Metabolites

**Figure 3:**
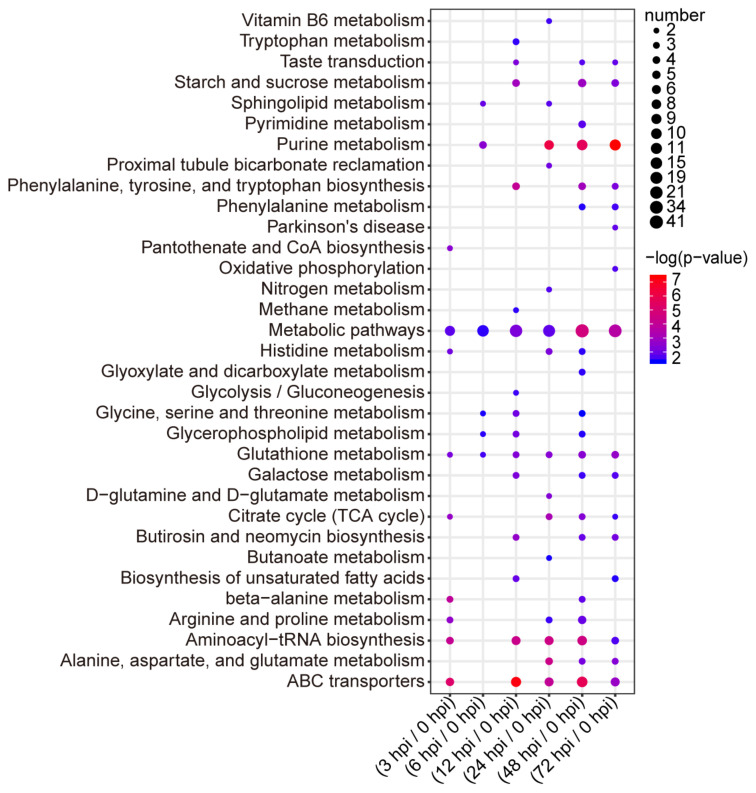
Pathway analysis of differential metabolites at 3, 6, 12, 24, 48, and 72 h post BmNPV infection.

The metabolites were located in the KEGG database and used to determine the pathways involved in host response to BmNPV. In total, there were 262 differential metabolites, which participated in 33 pathways (Table S4). There were nine pathways enriched in BmE cells infected with BmNPV at 3 hpi. ABC transporters, aminoacyl–tRNA biosynthesis, beta-alanine metabolism, citrate cycle (TCA cycle), arginine and proline metabolism, pantothenate and CoA biosynthesis, glutathione metabolism, and histidine metabolism changed significantly. There were six pathways enriched in BmE cells infected with BmNPV at 6 hpi but only purine metabolism changed significantly. There were 15 pathways enriched in BmE cells infected with BmNPV at 12 hpi. ABC transporters, aminoacyl–tRNA biosynthesis, phenylalanine, tyrosine and tryptophan biosynthesis, starch and sucrose metabolism, butirosin and neomycin biosynthesis, galactose metabolism, and taste transduction pathways changed significantly. There were 15 pathways enriched in BmE cells infected with BmNPV at 24 hpi. Purine metabolism, aminoacyl–tRNA biosynthesis, alanine, aspartate and glutamate metabolism, ABC transporters, TCA cycle, glutathione metabolism, D-glutamine and D-glutamate metabolism, histidine metabolism, and proximal tubule bicarbonate reclamation changed significantly. There were 20 pathways enriched in BmE cells infected with BmNPV at 48 hpi. ABC transporters, purine metabolism, metabolic pathways, aminoacyl–tRNA biosynthesis, phenylalanine, tyrosine and tryptophan biosynthesis, starch and sucrose metabolism, glutathione metabolism, TCA cycle, alanine, and aspartate and glutamate metabolism changed significantly. There were 16 pathways enriched in BmE cells infected with BmNPV at 72 hpi. Purine metabolism, metabolic pathways, ABC transporters, glutathione metabolism, alanine, aspartate and glutamate metabolism, starch and sucrose metabolism, phenylalanine, tyrosine and tryptophan biosynthesis, and butirosin and neomycin biosynthesis changed significantly (Figure 3).

**Figure 4:**
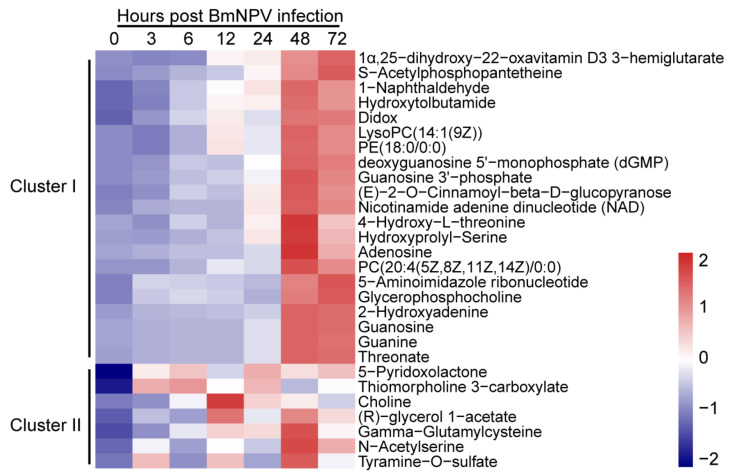
Hierarchical cluster analysis and the heatmap of the metabolites continuously increased at different infection stages.

Next, we used the R package for reactome pathway analysis to identify enriched metabolic pathways and determined that all differential metabolites at each time point (*p* < 0.05) mapped known biological processes (Figure 4). We observed that purine metabolic pathways, ammonia acyl transfer RNA signaling pathways, and ABC signaling pathways in different times continued to be the most greatly affected. These data suggest that these pathways are important in BmNPV infection.

### 2.4. Patterns of Metabolites in BmE Cells Infected with BmNPV at Different Times

To compare the metabolites that changed continuously at different times, the metabolites upregulated at three or more time points were screened and used for hierarchical cluster analysis. Heat map and abundance analysis showed the differential metabolites could be divided into two clusters. Cluster I contained mainly metabolites that were upregulated significantly at 48 and 72 hpi; Cluster II contained metabolites that were upregulated post infection with BmNPV at discontinuous hpi. Then, metabolites including GPC [22], 2-OH-Ade [23,24], γ-GC [25,26], 4-hydrotobutamide [27], and 5-pyridoxolactone [28] that were upregulated at the 4 hpi time point were selected as candidate metabolites that may be important in BmNPV infection.

Activated choline metabolism is a hallmark of carcinogenesis and tumor progression, such that the levels of glycerophosphocholine and phosphocholine are elevated in all types of cancer tests. Altered glycerophosphocholine (GPC) interacts with lipid and glucose metabolic pathways in cancer [22]. The 2-hydroxyadenine (2-OH-Ade) molecule is formed by hydroxyl radical attack on DNA bases and shows human genotoxicity. It may be the source of the mutations induced by reactive oxygen species [23]. A recent study revealed isoguanosine is not formed by oxidative stress, but rather that it could be formed by a more specific reaction such as, e.g., a post-transcriptional modification in RNA that may possess novel and unknown functions [24]. Gamma-glutamylcysteine (γ-GC) is an intermediate dipeptide of the GSH synthesis pathway and has anti-inflammatory properties. It represents a relatively unexplored option for sepsis treatment [25]. Supplementation with γ-GC lessens oxidative stress, brain inflammation, and amyloid pathology and improves spatial memory in a murine model of AD [26]. Hydroxytolbutamide (4-hydroxy tolbutamide) is a hydroxylation byproduct of tolbutamide. Tolbutamide stimulates the secretion of insulin by the pancreas [27]. The molecule 5-pyridoxolactone (a-pyracin) belongs to the class of organic compounds known as pyridinecarboxylic acids. Both 5-pyridoxolactone and 4-pyridoxolactone are formed by dehydrogenation of pyridoxal or isopyridoxal during the bacterial degradation of vitamin B6 by Pseudomonas MA-1 and Arthrobacter Cr-7, respectively. They are hydrolyzed to the corresponding acids by distinct inducible lactonases [28]. Whether these endogenous small molecules play roles in the host response to BmNPV infection has not been reported.

### 2.5. 5-Pyridoxolactone Is an Important Antiviral Molecule in Host

**Figure 5:**
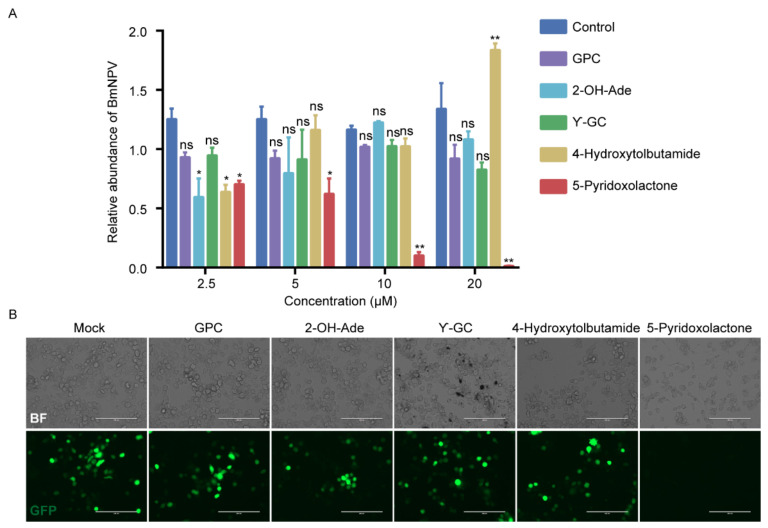
The 5-pyridoxolactone significantly inhibits the proliferation of BmNPV. (**A**) BmE cells were treated with 0, 2.5, 5, 10, and 20 μM GPC, 2-OH-Ade, γ-GC, 4-hydroxytobutamide, 5-pyridoxolactone, and BmNPV at MOI of 1, separately, and total genomes were extracted and relative viral DNA level was determined by qPCR at 48 hpi. (**B**) Fluorescence microscopy (GFP) of 20 μM GPC, 2-OH-Ade, γ-GC, 4-hydroxytobutamide, and 5-pyridoxolactone, separately, treated together with BmNPV cells or only BmNPV cells (scale bar = 200 μm). The data are displayed as mean ± SD of three independent experiments. Statistically significant differences between the mean values were determined by Student’s *t*-test (* *p* < 0.05, ** *p* < 0.01, ns—no significant difference).

To analyze the function of candidate metabolites screened from the heatmap, we evaluated whether increases in GPC, 2-OH-Ade, γ-GC, 4-hydrotobutamide, and 5-pyridoxolactone protected cells from viral infection. BmE cells were infected with BmNPV-GFP at a multiplicity of infection (MOI) of 1. Viral titer measurements showed that increasing the amounts of GPC, 2-OH-Ade, and γ-GC did not significantly affect the replication of BmNPV in BmE cells, while 4-hydrotobutamide inhibited the infection of BmNPV at 2.5 μM and promoted the proliferation of BmNPV at 20 μM. The increased dose of 5-pyridoxolactone, a vitamin B6 metabolite, decreased the viral DNA imported to the cells at 48 hpi compared with the control. The level of BmNPV DNA decreased significantly, with increasing 5-pyridoxolactone in a dose-dependent manner (Figure 5A). Fluorescence microscopy also showed that virus production decreased in 5-pyridoxolactone-treated cells (Figure 5B).

To exclude the possibility that the toxicity of 5-pyridoxolactone decreased proliferation of BmNPV, we treated groups of BmE cells with 0, 2.5, 5, 10, and 20 μM for 48 h separately. A CCK-8 assay of BmE cells treated with 2.5, 5, and 10 μM of 5-pyridoxolactone for 48 h showed no increased absorbance at 450 nm compared with the control (0 μM). On the basis of the results of the LIVE/DEAD cell dyeing assays, the rate of dead cells in BmE cells treated with 5-pyridoxolactone at 20 μM was not significantly increased compared with untreated cells (Figure S2B). These results suggest that 5-pyridoxolactone has an antiviral effect in host cells.

### 2.6. 5-Pyridoxolactone May Suppress the Invasion of BmNPV

**Figure 6:**
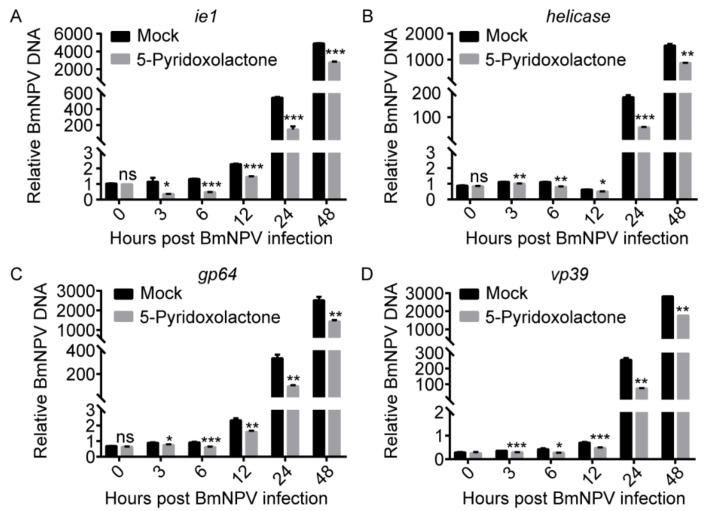
The 5-pyridoxolactone significantly inhibits the expression of BmNPV genes at different infection stages. (**A**–**D**) Expression of BmNPV ie1 (**A**), helicase (**B**), gp64 (**C**), and vp39 (**D**) in BmE cells treated with 10 μM 5-pyridoxolactone and BmNPV, separately or together, at 48 hpi. Statistically significant differences between the mean values were determined by Student’s *t*-test (* *p* < 0.05, ** *p* < 0.01, *** *p* < 0.001, ns—no significant difference).

The BmNPV genome encodes 136 genes, including essential and non-essential genes [29]. The expression of these genes follows an ordered time-level model, including four phases: very early (0–4 hpi), late early (5–7 hpi), late (8–18 hpi), and very late (>18 hpi) [30]. ie1 [31] of NPV is an essential gene in the very early stage, Helicase [32] is an essential gene in the late early stage, and GP64 [33,34] and VP39 [35] are essential genes in the late infection stage. To further analyze the period when 5-pyridoxolactone was active in inhibition of BmNPV infection, we collected BmE cells post BmNPV infection at 0, 3, 6, 12, 24, and 48 h and determined the gene expression of Ie1, Helicase, GP64, and VP39 (Figure 6). The proliferation of BmNPV was significantly suppressed from 3 to 48 hpi, suggesting that 5-pyridoxolactone decreased the invasion of BmNPV.

## 3. Discussion

Many studies have evaluated genomic, transcriptomic, and proteomic levels to study antiviral genes [36,37,38,39], but there are few metabolomics studies that have screened for small molecules in the host to inhibit the proliferation of BmNPV. We collected samples from cells infected with BmNPV at different times for metabolite extraction. LC-MS/MS analysis revealed that the number of differential metabolites in BmE cells increased with the infection time and the number of upregulated metabolites increased significantly. However, the downregulated metabolites showed a trend of increasing during the early infection stage and then decreasing. The pathways enriched by the differential metabolites were analyzed. ABC transporter, aminoacyl transport RNA, and purine metabolic signaling pathways were significantly enriched. Hierarchical cluster analysis and the heatmap of the identified metabolites from the BmNPV-infected BmE cells showed GPC, 2-OH-Ade, γ-GC, 4-hydrotobutamide, and 5-pyridoxolactone were continuously upregulated post BmNPV infection. We exposed BmE cells to BmNPV and GPC, 2-OH-Ade, γ-GC, 4-hydrotobutamide, and 5-pyridoxolactone separately, or BmNPV only. Proliferation of BmNPV was suppressed only in 5-pyridoxolactone-treated cells and this occurred in a dose-dependent manner. Finally, the inhibition of proliferation of BmNPV from 3 to 72 hpi suggests that 5-pyridoxolactone plays an important role in the early-infection stage of BmNPV.

In this study, we identified the intracellular metabolites of BmE cells infected with BmNPV at different times. Although multiple metabolic signaling pathways were enriched during BmNPV infection, ABC transporter, aminoacyl–transport RNA, and purine metabolism changed significantly at almost all infection stages. Among them, the ABC transporter pathway has been identified in multiple proteome and transcriptome studies [9,40]. In previous studies, knockout of the V-ATPase subunit gene BMgn016795A of the ABC transporter pathway resulted in a significant decrease in proliferation of BmNPV in BmE cells [8]. The endosomal acidification mediated by this gene may play a role in the process of BmNPV entering cells, although the mechanism is unknown. The aminoacyl–transporter RNA pathway was also identified, related to the antiviral mechanisms of the silkworm in transcriptome analysis of resistant and susceptible *B. mori* strains following BmNPV infection. However, the relationship between the purine metabolism and BmNPV infection in BmE cells requires further study.

This study showed that 5-pyridoxolactone was continuously increased in BmE cells infected with BmNPV and that this was beneficial to cells in response to BmNPV infection. The 5-pyridoxolactone is a product of the vitamin B6 degradation pathway. The 5-pyridoxolactone molecule is present in both insects and mammals, but its function is typically unknown. The discovery of 5-pyridoxolactone provides new research targets for the host–virus interaction. Functional analysis of this molecule may help reveal the defense mechanism used by the host against the virus and aid in the development of antiviral medicines.

## 4. Materials and Methods

### 4.1. Cells and BmNPV

BmE cells [41] and BmN4-SID1 cells [42] were maintained at 27 °C in Grace’s medium or IPL-41 medium supplemented with 10% (*v*/*v*) fetal bovine serum, penicillin, and streptomycin (Gibco, Carlsbad, CA, USA). BmNPV-GFP was used in this study. Viruses were propagated in BmE cells as previously described [43].

### 4.2. Sample Collection and Metabolite Extraction

BmE cells (3 × 10^6^) infected with BmNPV at 0, 3, 6, 12, 24, 48, and 72 hpi were collected in 100 μL of 40% (*v*/*v*) acetonitrile/40% (*v*/*v*) methanol/0.1 N formic acid methanol. This slurry was incubated for 30 min at −20 °C and concentrated at 4 °C for 15 min [21]. The supernatant was moved to a clean centrifuged tube, dried for 3 h in a vacuum concentrator, and then dissolved in 100 μL water.

### 4.3. LC-MS/MS Analysis

Ten microliters of each sample was injected into a Thermo Fisher Scientific Ultimate 3000 system (Thermo Fisher Scientific, Waltham, MA, USA) equipped with an Agilent Zorbax C18 column (3 µm, 2.1 × 150 mm, Agilent). The ionization source parameters were set as follows: positive mode; capillary temperature, 250 °C; and spray voltage, 2.3 kV. The flow phases used were A: 10 mM tributylamine plus 15 mM acetic acid in 97:3 water:methanol, and B: methanol. The gradient used was 99% A:1% B for 2.5 min; 80% A:20% B for 7.0 min; 35% A:65% B for 7.5 min; 5% A:95% B, for 9.01 min; and 99% A:1% B for 10 min. High-resolution accurate mass data were acquired in positive mode using a Thermo Scientific Q-Exactive Orbitrap mass spectrometer (Thermo Fisher Scientific) operated at a resolution of 70,000. The voltage of the electrospray source was set to 3.5 kV in the positive mode. Full scan MS spectra were acquired in a mass range from m/z 100 to 1000. The raw data were analyzed using the Component Extraction algorithm in SIEVE 2.0 software (Thermo Fisher Scientific) to detect the metabolites. The intensity threshold was set to 3,000,000, and three databases were chosen to identify the metabolites: Human Metabolome Database (HMDB), Metlin Metabolite Database, and Kyoto Encyclopedia of Genes and Genomes (KEGG). The m/z tolerance was set to 5 ppm for the database search.

### 4.4. Data Preprocessing and Analysis

Raw data files were converted into AIA data format and sent to MS online (https://xcmsonline.scripps.edu/index.php accessed on 20 June 2021) [1]. The metabolic peaks were identified by comparing their mass spectra with both raw data files and the NIST 2011 (version 2.0, National Institute of Standards and Technology, Gaithersburg, MD, USA) library and standard compounds. Only a relative score greater than 700 was considered to be a good match. Orthogonal partial least squares discriminant analysis (OPLS-DA) [44] was performed using SIMCA-P software (version 14.0) to obtain the separated trend of sample sets. To show patterns of metabolite abundance in different samples, a heat map was created by using MetaboAnalyst online software (http://www.metaboanalyst.ca/faces/home.xhtml accessed on 20 June 2021) [45]. To reveal the metabolic differences of BmE cells infected with BmNPV at 0, 3, 6, 12, 24, 48, and 72 hpi, the metabolites identified were subjected to differential analysis. Differential metabolites were identified following a previously described method [46], with only metabolites that changed ≥2 fold (enriched metabolites) or ≤0.5 (decreased metabolites) in relative ratios (*p*-value < 0.05) considered to be significantly altered.

### 4.5. Pathway Analysis

All the identified metabolites were submitted to the KEGG pathway database (http://www.kegg.jp/kegg/pathway.html accessed on 20 June 2021) to obtain the KEGG ID and the pathway ID. The KEGG ID and the pathways in which the metabolites are involved were obtained. Metabolites that were not identified in the database were not used for further analysis. Pathway enrichment was performed using the Metabolomics Pathway Analysis (MetPA) program as previously described [47].

### 4.6. Real-Time PCR

Total RNA was isolated from cells using the Total RNA Kit (Omega, Norcross, GA, USA) and reverse transcribed by GoScript TM Reverse Transcription System (Promega, Madison, WI, USA). Fluorescence real-time PCR analysis was performed using Ex Taq II (Takara, Kusatsu, Japan) on a 7500 fast Real-Time PCR System (Applied Biosystems, Bedford, MA, USA) with a program consisting of an initial denaturing step of 30 s at 95 °C and 40 amplification cycles consisting of 5 s at 95 °C, followed by 30 s at 60 °C.

### 4.7. Measurement of Viral DNA Load

At the indicated time points, BmNPV-infected BmE cells were harvested and suspended in PBS. Total DNA from each sample was prepared with a TaKaRa MiniBEST Universal Genomic DNA Extraction Kit (Takara, Kusatsu, Japan) according to manufacturer protocol. The viral DNA abundance of BmNPV was examined by the expression of GP64 gene. The silkworm GAPDH gene (BGIBMGA003186-TA: GAPDH) was used for normalization. Sequences of primers are listed in Table S1.

### 4.8. Cell Toxicity and Tests

BmE cells were treated with 0, 2.5, 5, 10, and 20 μM of 5-pyridoxolactone for 48 h. The viability of BmE cells was examined with a LIVE/DEAD Viability/Cytotoxicity Kit (Molecular Probes, Wokingham, UK) and a Cell Counting Kit-8 (Beyotime, Shanghai, China) as previously described [48].

### 4.9. Statistics

The results are expressed as the means ± s.e.m. Statistically significant differences between the mean values were determined by Student’s *t*-test (* *p* < 0.05, ** *p* < 0.01, *** *p* < 0.001, **** *p* < 0.0001). Silkworm experiments were performed in biological triplicate with the indicated number of silkworms per group. Cell culture experiments were collected from three or five independent cultures for each sample.

## Supplementary Materials

The following are available online at https://www.mdpi.com/article/10.3390/ijms22147423/s1.
