# Supplementary material for: The Antiviral Molecule 5-Pyridoxolactone Identified Post BmNPV Infection of the Silkworm, Bombyx mori
Source: Int J Mol Sci. 2021 Jul 10;22(14):7423. doi: 10.3390/ijms22147423 (PMC8307608; doi:10.3390/ijms22147423)
Supplement: Supplementary file 1 [file ijms-22-07423-s001.zip › supplementary files/Supplement Figure Legends.pdf]

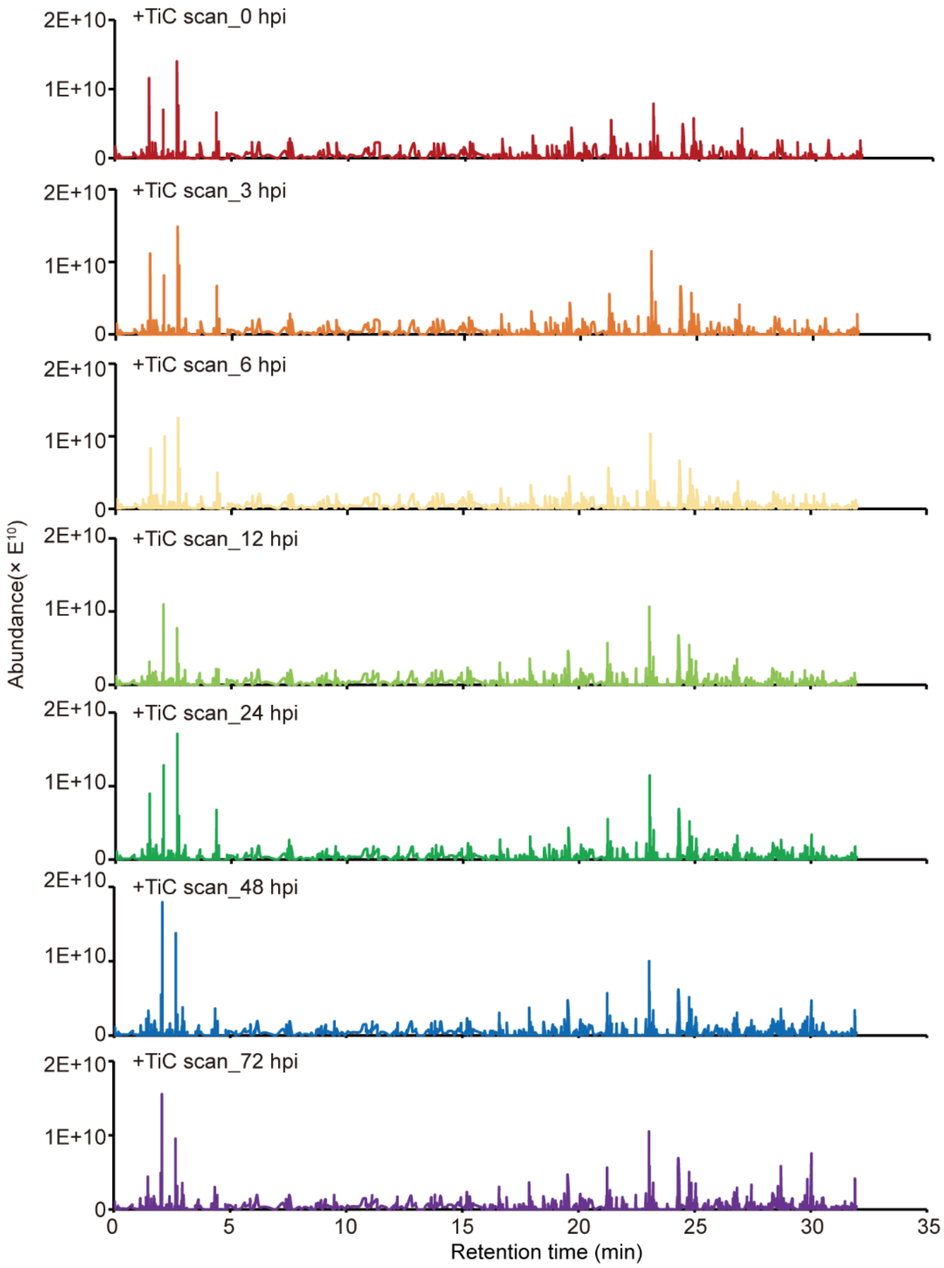

Figure S1. Total ion chromatograms (TICs) of BmE cells post BmNPV infection at different time.

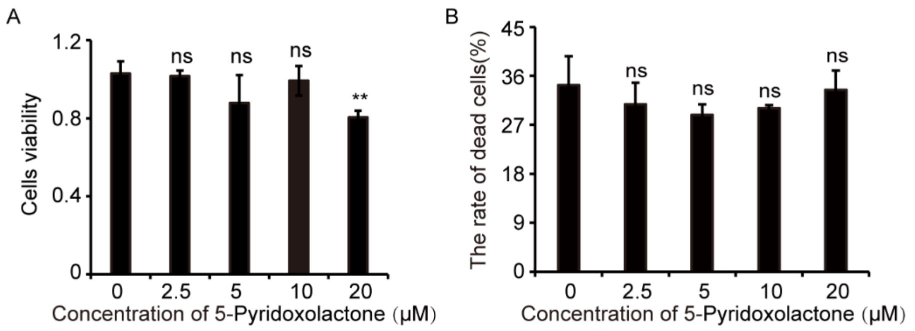

Figure S2. Cytotoxicity of 5-Pyridoxolactone in BmE and BmN4 cells. A. Cell variability of BmE cells treated with 0, 2.5, 5, 10, 20μM of 5-Pyridoxolactone was detected by CCK8; B. The rate of dead cells in BmE cells treated with 0, 2.5, 5, 10, 20μM of 5-Pyridoxolactone was detected by Live-Dead cell dyeing.
