# Supplementary material for: The Antiviral Molecule 5-Pyridoxolactone Identified Post BmNPV Infection of the Silkworm, Bombyx mori
Source: Int J Mol Sci. 2021 Jul 10;22(14):7423. doi: 10.3390/ijms22147423 (PMC8307608; doi:10.3390/ijms22147423)
Supplement: Supplementary file 1 [file ijms-22-07423-s001.zip › supplementary files/Table S1.pdf]

Table S1. Primers used in qRT-PCR

Gene	Forward primer	Reverse primer
Ie1	CACGGTCGCTTCAACTCAA	TGTCGTCGAAACGCATCAA
GP64	CCATCGTGGAGACGGACTA	CTCGCACTGCTGCCTGA
VP39	TAATGCCCGTGGGTATGG	GTTTGATGAGGTGGCTGTTGC
Helicase	AACACATGCCAAGCCGATAT	TCCCGACACCGTTGACC
GAPDH	CATTCCGCGTCCCTGTTGCTAAT	GCTGCCTCCTTGACCTTTTGC
