# Supplementary material for: The Antiviral Molecule 5-Pyridoxolactone Identified Post BmNPV Infection of the Silkworm, Bombyx mori
Source: Int J Mol Sci. 2021 Jul 10;22(14):7423. doi: 10.3390/ijms22147423 (PMC8307608; doi:10.3390/ijms22147423)
Supplement: Supplementary file 1 [file ijms-22-07423-s001.zip › supplementary files/Table S3.pdf]

Table S3_1. Differential metabolites identified in 3 hpi / 0 hpi.

metabolites	Fold change	VIP	*p* value
Uric acid	0.19	2.46	9.63E-03
Riboflavin cyclic-4',5'-phosphate	0.37	2.29	2.87E-12
beta-Estradiol 17-acetate	0.46	2.90	1.15E-03
Tyramine-O-sulfate	2.03	4.86	3.83E-04
(R)-glycerol 1-acetate	2.04	2.15	7.89E-03
5-Pyridoxolactone	2.15	5.57	6.84E-03
N-Acetylserine	2.22	5.90	8.11E-05
Pentadecanoic acid	2.48	2.52	1.87E-08
Thiomorpholine 3-carboxylate	2.55	7.06	3.82E-03
Glycerophosphocholine	2.84	3.46	3.92E-08
2-Carboxy-4-dodecanolide	2.86	2.18	3.94E-11
Pentadecanoic acid	2.98	2.53	6.50E-09
5-Aminoimidazole ribonucleotide	3.78	2.29	3.39E-07
Glycerophosphocholine	3.85	3.28	3.39E-08

Table S3_2. Differential metabolites identified in 6 hpi / 0 hpi.

metabolites	Fold change	VIP	*p* value
5,7,9,11,13-tetradecapentaenoic acid	0.30	2.38	5.19E-05
S-Acetyldihydrolipoamide-E	0.33	4.43	3.21E-05
L-Histidinol	0.34	2.46	4.53E-03
3E,5E-tridecadienoic acid	0.40	3.94	1.31E-04
PC(8:2(2E,4E)/8:2(2E,4E))	0.41	2.35	6.00E-09
N-hydroxy arachidonoyl amine	0.47	2.06	2.17E-09
2,3-Dinor-6-keto-PGF1a	0.48	3.42	2.87E-09
Hydroxytolbutamide	2.02	7.30	9.58E-07
2-hydroxy pelargonic acid	2.05	4.58	8.22E-05
Traumatic acid	2.15	2.78	4.06E-03
Gamma-Glutamylcysteine	2.27	3.43	9.37E-12
5-Pyridoxolactone	2.34	4.96	2.63E-03
Thiomorpholine 3-carboxylate	2.67	5.89	2.56E-03
Glycerophosphocholine	3.31	3.24	1.05E-09
1-hexadecylpyridinium	3.51	3.71	7.18E-04
Glycerophosphocholine	3.87	2.71	5.35E-07
2-Hydroxyadenine	4.14	3.81	9.20E-05
17-keto-7(Z),10(Z),13(Z),15(E),19(Z)-Docosapentaenoic Acid	9.57	3.02	6.24E-03

Table S3_3. Differential metabolites identified in 12 hpi / 0 hpi.

metabolites	Fold change	VIP	*p* value
PC(8:2(2E,4E)/8:2(2E,4E))	0.08	2.26	1.53E-13
cN-hydroxy arachidonoyl amine	0.08	2.10	6.96E-15
2,3-Dinor-6-keto-PGF1a	0.09	3.48	4.05E-15
7-oxo-11E-Tetradecenoic acid	0.09	2.05	1.08E-12
Alpha-Lactose	0.11	5.37	6.70E-11
Formetanate	0.11	2.59	4.20E-15
Tyramine glucuronide	0.13	2.02	3.33E-10
Melamine	0.15	2.14	1.33E-11
Caffeic acid 4-O-glucuronide	0.16	4.60	1.36E-09
Pantothenic acid	0.16	2.28	2.39E-12
5-Methyl-THF	0.16	5.23	3.45E-12
Indoleacetic acid	0.16	3.80	1.10E-09
4-Hydroxybenzaldehyde	0.17	2.58	1.68E-09
9Z,11E,13-Tetradecatrienal	0.17	3.67	1.56E-10
Glutathione	0.18	2.02	1.92E-14
a-L-Arabinofuranosyl-(1->3)-[a-L-arabinofuranosyl-(1r5)]-L-arabinose	0.19	2.72	3.38E-13
Pilocarpine	0.19	5.30	1.34E-11
(S)-a-Amino-2,5-dihydro-5-oxo-4-isoxazolepropanoic acid N2-glucoside	0.20	4.26	2.22E-15
5-Deoxykievitone hydrate	0.20	2.08	3.04E-12
5,10-Methylene-THF	0.20	3.26	8.27E-11
D-Lactose	0.21	3.16	4.15E-10
L-Tyrosine	0.22	2.45	4.66E-12
beta-Estradiol 17-acetate	0.23	2.23	7.39E-05
3-b-Galactopyranosyl glucose	0.24	2.45	1.09E-11
L-Tryptophan	0.26	2.74	8.11E-12
N-Acetyl-D-fucosamine	0.27	3.60	1.30E-10
Indole	0.27	2.38	1.86E-11
L-Tryptophan	0.27	10.33	1.36E-10
3-amino-2-naphthoic acid	0.27	12.26	1.45E-10
Indoleacrylic acid	0.27	2.19	6.13E-12
2-Oxosuberate	0.28	4.27	2.41E-10
Indole-3-carboxaldehyde	0.29	4.40	8.66E-11
Furocoumarinic acid glucoside	0.29	4.20	7.32E-12
Cellulose	0.30	2.31	1.25E-12
Ethyl Oxalacetate	0.30	4.01	1.23E-07
4-Methoxybenzyl O-(2-sulfoglucoside)	0.30	8.01	9.62E-11
1,5-Naphthalenediamine	0.30	2.33	2.08E-10
Sucrose	0.30	24.47	9.70E-12
4-Hydroxy-1-(3-pyridinyl)-1-butanone	0.32	12.67	1.42E-07
Phosphorylcholine	0.34	2.14	1.27E-10

Naptalam	0.36	9.48	1.73E-10
D-Mannitol	0.36	2.41	2.24E-10
3-(4-Methyl-3-pentenyl)thiophene	0.36	5.55	1.07E-09
L-Phenylalanine	0.36	17.64	7.85E-10
L-Tyrosine	0.36	7.74	4.56E-10
Benzofuran	0.38	2.06	4.60E-10
2-Hydroxycinnamic acid	0.38	2.97	6.07E-11
D-Glucose 6-phosphate	0.39	3.04	1.11E-11
4-O-Methyl-a-D-glucosyl-(1->2)-b-D-xylosyl-(1->4)-D-xylose	0.41	2.31	3.25E-09
2-Hydroxymethylserine	0.41	3.76	4.57E-10
Succinoadenosine	0.42	6.71	2.28E-10
Lactulose	0.43	18.12	8.98E-09
3,8-dimethyldec-7-en-1-yl trihydrogen diphosphate	0.44	2.75	2.45E-03
2-[3-Carboxy-3-(methylammonio)propyl]-L-histidine	0.44	3.55	6.07E-10
2,6-Dimethoxy-4-propylphenol	0.46	2.07	4.28E-07
L-Lysine	0.47	2.82	1.02E-10
N,N-dimethyl-Safingol	2.01	10.05	3.23E-05
Tyramine-O-sulfate	2.02	3.08	6.82E-07
Stearaldehyde	2.02	2.77	1.80E-04
1-Docosene	2.03	4.74	3.19E-05
Estra-1,3,5(10)-triene-3,6beta,17beta-triol triacetate	2.05	2.71	1.13E-03
3-Acrylamidopropyl trimethylammonium	2.10	3.15	4.65E-04
3-hydroxy-tetracosanoic acid	2.26	4.69	4.59E-05
4alpha-methyl-5alpha-cholestan-3beta-ol	2.27	2.42	4.61E-05
2-amino-14,16-dimethyloctadecan-3-ol	2.30	2.13	6.46E-05
N-Acetylserine	2.33	3.81	5.15E-05
Docosanoic acid	2.41	8.41	1.81E-05
N,N,N-trimethyl-sphingosine	2.42	4.13	2.02E-05
PE(18:0/0:0)	2.43	4.33	7.83E-06
LysoPC(14:1(9Z))	2.49	2.14	1.07E-05
Didox	2.58	2.30	1.14E-10
Hydroxytolbutamide	2.73	7.35	2.10E-09
2S-aminohexadecanoic acid	2.83	3.87	1.21E-04
Gamma-Glutamylcysteine	2.92	3.14	2.94E-06
Glycerophosphocholine	3.06	2.35	1.05E-11
Deamino-α-keto-demethylphosphinothricin	3.21	5.36	2.72E-05
Choline	3.45	3.45	6.31E-05
(R)-glycerol 1-acetate	4.44	2.78	3.33E-05
2-Hydroxyadenine	4.89	3.25	1.88E-05

Table S3_4. Differential metabolites identified in 24 hpi / 0 hpi.

metabolites	Fold change	VIP	*p* value
beta-Estradiol 17-acetate	0.26	2.37	1.77E-04
(±)-3-Hydroxynonanoic acid	0.33	2.33	8.47E-08
2,6-Dimethoxy-4-propylphenol	0.34	2.52	4.87E-08
Hexyl 2-furoate	0.35	2.32	4.24E-08
S-Acetyldihydrolipoamide-E	0.46	3.32	1.62E-04
nicotinate beta-D-ribonucleotide	2.04	3.52	2.63E-06
Hydroxyprolyl-Serine	2.04	2.15	9.24E-09
1-Naphthaldehyde	2.10	2.22	9.26E-08
Threonate	2.16	8.10	1.25E-11
Nicotinamide adenine dinucleotide (NAD)	2.19	2.65	1.37E-10
Inosine	2.22	3.36	8.60E-10
Choline	2.24	2.79	1.26E-07
Guanosine 3'-phosphate	2.28	2.36	8.38E-10
Acetylcarnitine	2.30	4.92	2.17E-17
13,14-dihydro-15-keto-tetranor PGE2	2.35	2.37	4.25E-10
5-Pyridoxolactone	2.43	4.40	1.85E-03
Thiomorpholine 3-carboxylate	2.49	4.67	6.20E-03
3'-UMP	2.50	2.14	6.93E-09
Guanine	2.57	4.70	1.15E-09
Guanosine	2.61	3.85	3.86E-09
Hydroxytolbutamide	2.76	8.03	1.35E-06
4-Hydroxy-L-threonine	2.79	2.65	3.57E-04
S-Acetylphosphopantetheine	2.85	2.77	7.63E-09
Gamma-Glutamylcysteine	2.85	3.44	2.13E-08
deoxyguanosine 5'-monophosphate (dGMP)	2.89	13.08	7.50E-10
(E)-2-O-Cinnamoyl-beta-D-glucopyranose	2.93	4.25	3.91E-10
L-DOPA 3'-glucoside	2.93	2.55	4.12E-11
Adenosine	3.22	2.32	3.37E-09
PE(14:0/22:5(4Z,7Z,10Z,13Z,16Z))	5.03	2.09	6.93E-03
2-Hydroxyadenine	6.04	3.93	3.30E-04
Vaccenyl carnitine	6.34	2.09	9.43E-06
1α,25-dihydroxy-22-oxavitamin D3 3-hemiglutarate	13.20	2.33	1.87E-03
7-Hydroxyenterolactone	15.20	2.23	1.79E-10

Table S3_5. Differential metabolites identified in 48 hpi / 0 hpi.

metabolites	Fold change	VIP	*p* value
beta-Estradiol 17-acetate	0.03	2.27	1.01E-05
PC(8:2(2E,4E)/8:2(2E,4E))	0.04	2.05	1.05E-13
2,3-Dinor-6-keto-PGF1a	0.06	3.16	3.09E-15
Formetanate	0.09	2.35	3.53E-15
Alpha-Lactose	0.17	4.63	1.73E-10
Caffeic acid 4-O-glucuronide	0.18	4.05	1.56E-09
Indoleacetic acid	0.18	3.36	1.19E-09
(S)-a-Amino-2,5-dihydro-5-oxo-4-isoxazolepropanoic acid N2-glucoside	0.19	3.82	5.05E-14
D-Glucoside	0.20	2.20	8.09E-08
9Z,11E,13-Tetradecatrienal	0.21	3.21	2.21E-10
D-Lactose	0.22	2.81	5.82E-10
Pilocarpine	0.24	4.60	3.69E-11
N-stearoyl taurine	0.24	3.08	1.49E-07
Citric acid	0.24	2.30	4.10E-04
Cellulose	0.26	2.12	3.80E-13
L-Tyrosine	0.28	2.11	2.21E-11
Furocoumarinic acid glucoside	0.30	3.74	7.03E-12
2,6-Dimethoxy-4-propylphenol	0.30	2.12	2.73E-08
Sucrose	0.31	21.81	1.05E-11
4-Hydroxybenzaldehyde	0.32	2.05	2.53E-06
Indole	0.33	2.04	7.73E-11
3-b-Galactopyranosyl glucose	0.33	2.06	4.42E-11
L-Tryptophan	0.34	2.32	3.63E-11
Naptalam	0.35	8.51	1.49E-10
N-Acetyl-D-fucosamine	0.37	2.98	1.03E-09
L-Tryptophan	0.37	8.55	1.04E-09
α-D-Glucose	0.37	3.07	1.54E-13
3-amino-2-naphthoic acid	0.38	10.11	1.24E-09
2-Oxosuberate	0.38	3.52	1.93E-09
Indole-3-carboxaldehyde	0.39	3.64	7.27E-10
Succinoadenosine	0.40	6.10	5.25E-10
D-Glucose 6-phosphate	0.40	2.69	2.59E-11
Lactulose	0.41	16.39	1.53E-08
Ethyl Oxalacetate	0.41	3.25	8.23E-07
5-Methyl-THF	0.43	3.85	3.09E-10
3,8-dimethyldec-7-en-1-yl trihydrogen diphosphate	0.44	2.44	2.59E-03
4-Hydroxy-1-(3-pyridinyl)-1-butanone	0.45	10.13	1.19E-06
Spermine	0.45	5.08	5.58E-09
5,10-Methylene-THF	0.46	2.39	3.14E-08
Dihydro Isorescinnamine	2.04	3.18	3.26E-04
Zalcitabine monophosphate	2.04	6.53	4.11E-06

Brunfelsamidine	2.05	2.22	4.07E-05
Choline	2.06	2.02	6.14E-05
Erucic acid	2.08	4.63	1.89E-05
N,N,N-trimethyl-sphingosine	2.10	3.23	2.13E-05
Docosanoic acid	2.11	6.64	1.52E-05
(5-Phenyl-1,2,4-triazol-3-yl)urea	2.12	2.56	2.44E-12
Palmitaldehyde	2.13	4.73	1.37E-05
Adenine	2.16	2.13	1.71E-10
L-Proline	2.16	7.98	3.40E-05
(4E,d14:1) sphingosine	2.17	4.12	5.16E-07
2-hydroxy-15Z-tetracosenoic acid	2.19	2.79	8.45E-06
5-Pyridoxolactone	2.20	3.22	5.42E-03
Calafatimine	2.21	2.18	7.95E-05
2-butyl-6-pentylpiperidine	2.22	2.83	5.96E-06
2-Hexylbenzothiazole	2.33	2.82	2.37E-13
Sphingosine	2.40	2.26	3.42E-06
13,14-dihydro-15-keto-tetranor PGE2	2.45	2.02	1.61E-11
Topaquinone	2.56	7.84	1.45E-14
4-Hydroxyxanthone	2.57	2.18	8.95E-14
Tyramine-O-sulfate	2.58	3.41	8.25E-07
L-Carnitine	2.58	4.82	8.35E-11
11E,13-Tetradecadienal	2.62	5.55	1.12E-07
Tetrahydrodipicolinate	2.65	2.21	5.90E-15
L-2-Amino-6-oxoheptanedioate	2.66	5.87	2.05E-15
9Z,12Z,15Z-Octadecatrienal	2.68	2.20	1.23E-07
Glymidine	2.70	5.47	7.16E-17
Adenosine 3'-monophosphate	2.71	2.95	3.17E-13
Glutathione	2.72	26.76	5.84E-17
Inodxyl glucuronide	2.77	3.00	1.30E-14
L-DOPA 3'-glucoside	2.78	2.01	4.07E-11
PE(18:1(9Z)/0:0)	2.80	6.39	4.82E-07
Phosphocreatinine	2.82	2.53	2.56E-10
PE(17:2(9Z,12Z)/0:0)	2.85	3.27	4.10E-07
1-Naphthaldehyde	2.93	2.43	7.85E-16
L-L-Homoglutathione	2.94	3.46	4.79E-15
4-Hydroxy-L-threonine	2.94	5.82	8.81E-11
PE(16:0/0:0)	2.94	3.44	2.85E-07
Didox	2.96	2.29	1.93E-13
Didox	3.09	2.18	1.52E-12
Inosine	3.14	4.94	3.18E-13
Uridine diphosphate-N-acetylglucosamine	3.23	2.91	1.90E-13
PC(18:1(9Z)/0:0)	3.28	5.55	7.06E-08
L-Glutamate	3.29	7.97	1.55E-13
LysoPE(20:2(11Z,14Z)/0:0)	3.32	2.91	9.39E-08

3'-UMP	3.35	2.21	6.50E-12
LysoPC(16:1(9Z))	3.39	3.73	2.74E-08
Succinic acid	3.48	4.73	7.85E-09
Nicotinamide adenine dinucleotide (NAD)	3.49	3.13	1.36E-13
Uracil	3.49	3.23	4.67E-12
nicotinate beta-D-ribonucleotide	3.59	4.48	9.12E-06
S-(Hydroxymethyl)glutathione	3.65	4.66	2.69E-13
Hydroxyprolyl-Serine	3.66	2.81	1.55E-08
Uridine diphosphate-N-acetylglucosamine	3.71	3.14	3.26E-13
Phosphocreatinine	3.82	2.23	2.73E-10
PE(18:0/0:0)	3.88	5.56	2.30E-07
Guanosine	4.03	2.39	1.37E-10
LysoPC(14:1(9Z))	4.06	2.78	2.14E-07
PC(18:1(6Z)/0:0)	4.16	6.97	3.01E-08
Gamma-Glutamylcysteine	4.18	3.67	3.48E-08
N-Acetylserine	4.22	5.40	9.01E-06
LysoPE(0:0/20:2(11Z,14Z))	4.23	3.73	3.60E-08
(R)-glycerol 1-acetate	4.25	2.27	8.98E-04
Hydroxytolbutamide	4.33	9.16	8.64E-13
PC(20:4(5Z,8Z,11Z,14Z)/0:0)	4.42	2.84	2.46E-08
S-Acetylphosphopantetheine	4.54	3.16	3.69E-17
PE(18:1(9Z)/0:0)	4.57	2.53	1.28E-09
LysoPC(16:0)	4.61	4.95	1.04E-09
UDP-N-acetyl-D-galactosamine	4.63	3.09	2.37E-11
Xanthine-8-carboxylate	4.70	3.28	2.80E-11
Guanosine 3'-phosphate	4.85	3.36	3.78E-14
4-Hydroxy-L-threonine	4.87	3.32	2.00E-05
Adenylosuccinate	4.88	4.47	2.15E-11
Inosine 5'-monophosphate (IMP)	5.07	2.34	6.54E-10
(E)-2-O-Cinnamoyl-beta-D-glucopyranose	5.10	5.08	9.03E-12
LysoPC(18:3(6Z,9Z,12Z))	5.18	2.06	1.11E-09
D-Glycerol 1-phosphate	5.20	2.96	1.74E-13
deoxyguanosine 5'-monophosphate (dGMP)	5.69	16.89	3.37E-12
PG(18:2(9Z,12Z)/0:0)	5.72	2.86	3.92E-03
Xanthine	5.85	3.96	8.83E-12
3-Methylthiopropyl-desulfoglucosinolate	6.18	2.19	2.25E-11
Threonate	6.23	14.13	2.32E-17
Inosine	6.26	5.74	3.53E-16
Glycerophosphocholine	7.10	2.72	2.17E-10
D-Glycerol 1-phosphate	7.52	2.53	5.75E-15
Guanine	8.27	8.31	2.17E-16
Glycerophosphocholine	8.58	4.02	4.66E-14
p-Cresol glucuronide	8.66	2.27	7.85E-18
N-(2'-(4-benzenesulfonamide)-ethyl) arachidonoyl amine	8.81	2.65	7.82E-03

Guanosine	8.93	7.03	6.13E-18
S-Adenosylmethionine	10.03	2.40	1.47E-13
5-Aminoimidazole ribonucleotide	10.38	2.38	3.70E-11
Cytosine	10.50	3.07	2.73E-15
Adenosine	15.34	4.84	2.84E-13
Deoxyadenosine monophosphate	16.52	3.76	2.01E-16
Cytidine	20.16	3.09	7.45E-15
1α,25-dihydroxy-22-oxavitamin D3 3-hemiglutarate	23.15	2.64	5.37E-04
UDP-N-acetyl-3-(1-carboxyvinyl)-D-glucosamine	23.35	2.29	1.66E-11
2-Hydroxyadenine	30.34	8.29	3.01E-13

Table S3_6. Differential metabolites identified in 72 hpi / 0 hpi.

metabolites	Fold change	VIP	*p* value
beta-Estradiol 17-acetate	0.04	2.22	1.09E-05
4-Hydroxybenzaldehyde	0.13	2.31	1.18E-07
2,3-Dinor-6-keto-PGF1a	0.16	2.95	6.41E-14
Formetanate	0.20	2.18	1.34E-13
(S)-a-Amino-2,5-dihydro-5-oxo-4-isoxazolepropanoic acid N2-glucoside	0.22	3.69	8.43E-14
Indoleacetic acid	0.23	3.21	2.49E-09
Caffeic acid 4-O-glucuronide	0.23	3.85	3.63E-09
Alpha-Lactose	0.25	4.32	5.07E-10
D-Glucoside	0.26	2.09	1.61E-07
2,6-Dimethoxy-4-propylphenol	0.27	2.14	1.66E-08
D-Lactose	0.28	2.67	1.16E-09
Pilocarpine	0.30	4.36	1.15E-10
9Z,11E,13-Tetradecatrienal	0.34	2.88	6.25E-09
L-Tryptophan	0.36	2.25	7.83E-11
5-Methyl-THF	0.37	3.98	5.08E-11
α-D-Glucose	0.38	3.01	1.72E-11
N-Acetyl-D-fucosamine	0.40	2.87	1.74E-09
L-Tryptophan	0.40	8.22	2.00E-09
Furocoumarinic acid glucoside	0.41	3.38	1.94E-10
3-amino-2-naphthoic acid	0.41	9.68	2.55E-09
Sucrose	0.41	19.71	3.01E-10
2-Oxosuberate	0.42	3.36	4.26E-09
Indole-3-carboxaldehyde	0.42	3.49	1.44E-09
Naptalam	0.43	7.83	2.41E-09
5,10-Methylene-THF	0.45	2.37	6.69E-09
Ethyl Oxalacetate	0.45	3.10	1.70E-06
Succinoadenosine	0.47	5.58	4.38E-08
5-(2-Hydroxyethyl)-4-methylthiazole acetate	0.48	2.28	9.08E-12
Lactulose	0.48	15.16	1.47E-07
Nicotinic acid	0.48	2.35	5.09E-13
4-Hydroxy-1-(3-pyridinyl)-1-butanone	0.49	9.59	9.20E-06
3-(4-Methyl-3-pentenyl)thiophene	0.49	4.34	1.49E-08
2-Acetylpyrazine	0.50	4.22	6.40E-09
L-Phenylalanine	0.50	13.74	1.03E-08
N2-Acetyl-L-aminoadipate	2.00	2.49	3.49E-11
3-hydroxy-tetracosanoic acid	2.02	3.82	3.86E-04
Calafatimine	2.06	2.06	1.84E-04
Erucic acid	2.07	4.65	2.26E-04
Adenine	2.09	2.03	4.40E-11
Hexyl dodecanoate	2.14	9.31	1.27E-05
2-hydroxy-15Z-tetracosenoic acid	2.15	2.74	2.64E-05

2-butyl-6-pentylpiperidine	2.26	2.91	3.87E-07
2-Hexylbenzothiazole	2.29	2.73	1.03E-12
(5-Phenyl-1,2,4-triazol-3-yl)urea	2.30	2.73	7.94E-13
5-Pyridoxolactone	2.36	3.40	2.43E-03
Glymidine	2.39	4.88	1.19E-14
LysoPE(20:2(11Z,14Z)/0:0)	2.41	2.26	6.30E-07
Glutathione	2.41	23.89	1.05E-14
PC(18:1(9Z)/0:0)	2.42	4.35	8.12E-07
LysoPC(16:1(9Z))	2.42	2.87	2.40E-08
Inodxyl glucuronide	2.46	2.69	1.00E-12
PE(16:0/0:0)	2.47	2.97	3.98E-06
Hydroxyprolyl-Serine	2.48	2.08	6.33E-12
Succinic acid	2.49	3.65	1.00E-10
PE(18:1(9Z)/0:0)	2.51	5.85	3.08E-06
PE(17:2(9Z,12Z)/0:0)	2.56	3.00	2.57E-06
Gamma-Glutamylcysteine	2.60	2.59	2.66E-11
1-Naphthaldehyde	2.62	2.19	5.54E-14
Adenosine 3'-monophosphate	2.73	2.93	3.95E-11
Topaquinone	2.86	8.46	1.74E-12
L-L-Homoglutathione	2.86	3.35	1.04E-13
L-Glutamate	2.87	7.11	1.62E-13
4-Hydroxyxanthone	2.88	2.36	1.81E-12
Tetrahydrodipicolinate	2.95	2.37	3.20E-12
L-2-Amino-6-oxoheptanedioate	2.96	6.31	1.93E-12
Palmitoleoyl-EA	3.03	2.19	1.43E-09
Didox	3.03	2.31	1.46E-13
Inosine	3.04	4.77	3.62E-13
Didox	3.06	2.14	1.19E-14
Nicotinamide adenine dinucleotide (NAD)	3.13	2.86	1.19E-12
N-Acetylserine	3.17	4.35	3.92E-05
LysoPC(16:0)	3.18	3.81	3.26E-08
L-Carnitine	3.20	5.62	1.25E-11
Xanthine-8-carboxylate	3.33	2.57	1.68E-10
Uracil	3.37	3.12	2.20E-13
PC(18:1(6Z)/0:0)	3.41	6.05	5.73E-08
LysoPE(0:0/20:2(11Z,14Z))	3.45	3.24	4.52E-08
PE(18:0/0:0)	3.47	5.13	1.62E-06
4-Hydroxy-L-threonine	3.61	2.46	6.04E-03
LysoPC(14:1(9Z))	3.63	2.57	1.60E-06
PC(20:4(5Z,8Z,11Z,14Z)/0:0)	3.64	2.48	1.75E-08
Guanosine	3.65	2.21	9.18E-11
S-(Hydroxymethyl)glutathione	3.71	4.60	9.66E-06
Uridine diphosphate-N-acetylglucosamine	3.75	3.20	4.76E-14
Oleamide	3.81	7.07	1.70E-07

Hydroxytolbutamide	3.87	8.39	4.41E-12
Guanosine 3'-phosphate	4.19	3.02	1.04E-13
Xanthine	4.22	3.19	2.44E-12
(E)-2-O-Cinnamoyl-beta-D-glucopyranose	4.27	4.46	3.58E-10
ADP	4.47	2.46	4.18E-03
Uridine diphosphate-N-acetylglucosamine	4.53	3.54	4.17E-14
D-Glycerol 1-phosphate	4.62	2.71	2.98E-12
Inosine 5'-monophosphate (IMP)	5.12	2.33	4.79E-11
p-Hydroxynorpropoxyphene	5.17	2.23	2.63E-14
deoxyguanosine 5'-monophosphate (dGMP)	5.19	15.79	2.88E-14
S-Acetylphosphopantetheine	5.32	3.44	3.63E-14
Adenylosuccinate	5.35	4.68	1.32E-09
3-Methylthiopropyl-desulfoglucosinolate	5.66	2.05	3.66E-13
UDP-N-acetyl-D-galactosamine	5.76	3.50	6.56E-13
Threonate	5.99	13.64	8.91E-15
Inosine	6.32	5.70	4.45E-14
D-Glycerol 1-phosphate	6.57	2.31	1.23E-15
Guanine	7.82	7.95	2.93E-14
PS(17:1(9Z)/0:0)	7.94	2.12	9.67E-03
PG(18:2(9Z,12Z)/0:0)	8.04	3.80	9.38E-03
p-Cresol glucuronide	8.34	2.20	1.16E-13
N-(2'-(4-benzenesulfonamide)-ethyl) arachidonoyl amine	8.37	2.02	9.91E-03
Glycerophosphocholine	8.51	2.98	1.89E-11
Guanosine	8.64	6.81	8.75E-14
Adenosine	9.10	3.59	1.19E-11
S-Adenosylmethionine	9.31	2.27	1.24E-13
Glycerophosphocholine	9.62	4.24	2.35E-17
Cytosine	11.37	3.16	4.59E-13
5-Aminoimidazole ribonucleotide	12.23	2.58	2.00E-12
N-(2'-(4-benzenesulfonamide)-ethyl) arachidonoyl amine	12.49	3.56	8.36E-03
PG(15:0/0:0)	12.60	2.01	8.53E-03
Deoxyadenosine monophosphate	16.02	3.64	2.49E-11
Cytidine	22.36	3.22	2.39E-14
1α,25-dihydroxy-22-oxavitamin D3 3-hemiglutarate	27.88	2.94	4.03E-03
2-Hydroxyadenine	28.84	7.98	1.83E-12
2E,4E-tetradecadienoic acid	29.23	2.24	5.15E-10
