# Supplementary figures and images for: The Antiviral Molecule 5-Pyridoxolactone Identified Post BmNPV Infection of the Silkworm, Bombyx mori
Source: Int J Mol Sci. 2021 Jul 10;22(14):7423. doi: 10.3390/ijms22147423 (PMC8307608; doi:10.3390/ijms22147423)

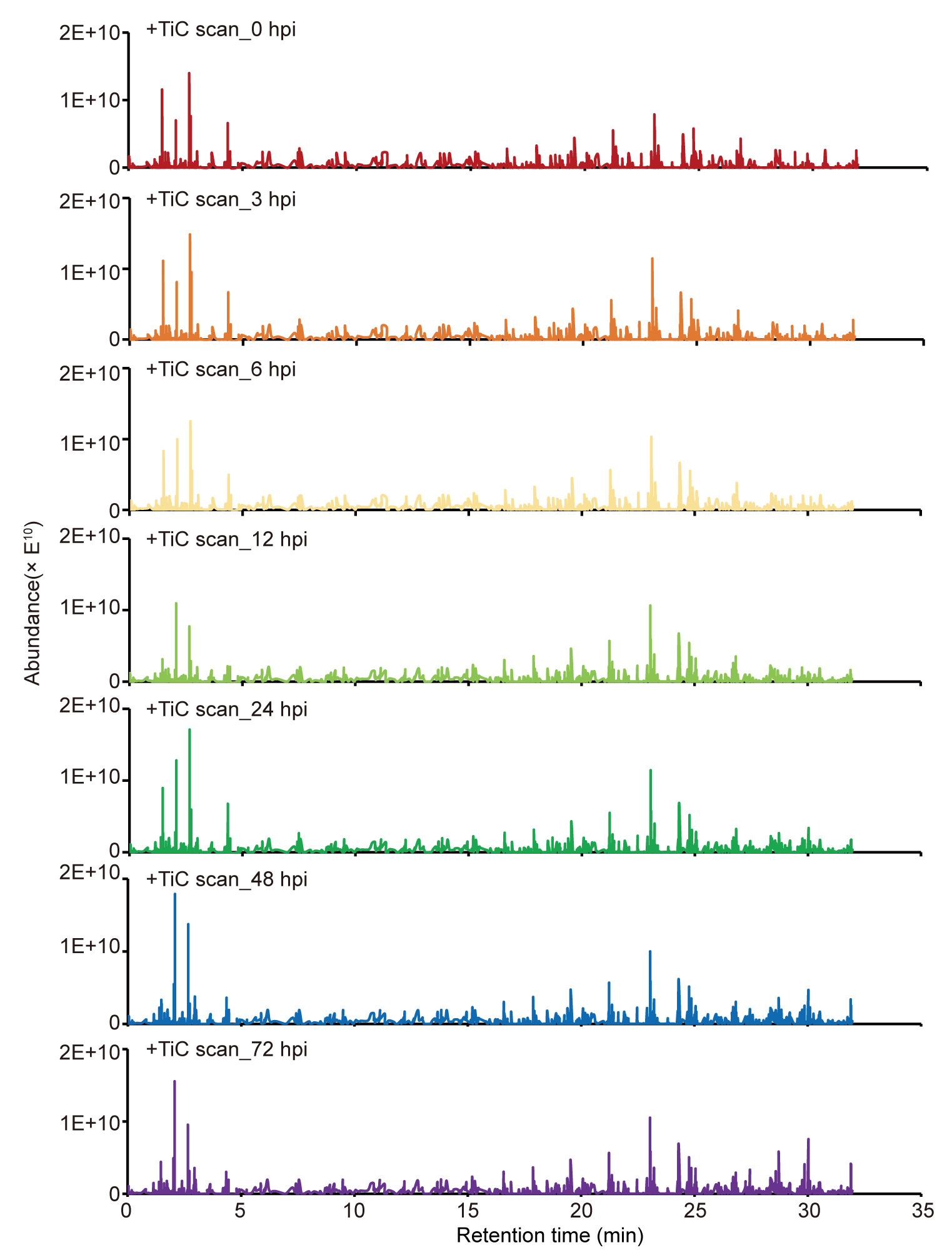

Supplement: Supplementary file 1 [file ijms-22-07423-s001.zip › supplementary files/Figure S1.tif]

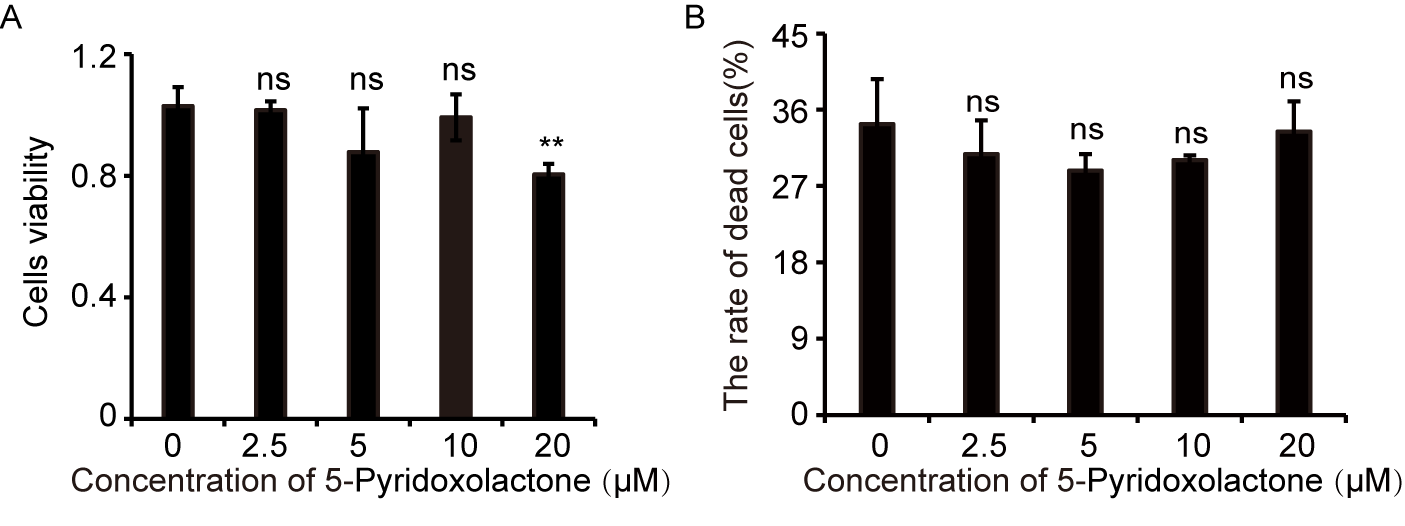

Supplement: Supplementary file 1 [file ijms-22-07423-s001.zip › supplementary files/Figure S2.tif]
